# Manual Compression versus Suture-Mediated Closure Device Technique for VA-ECMO Decannulation

**DOI:** 10.1155/2022/9915247

**Published:** 2022-03-18

**Authors:** Clemens Scherer, Christopher Stremmel, Enzo Lüsebrink, Thomas J. Stocker, Konstantin Stark, Carmen Schönegger, Antonia Kellnar, Jan Kleeberger, Maja Hanuna, Tobias Petzold, Sven Peterss, Daniel Braun, Jörg Hausleiter, Christian Hagl, Steffen Massberg, Martin Orban

**Affiliations:** ^1^Department of Medicine I, University Hospital, LMU, Munich, Germany; ^2^DZHK (German Centre for Cardiovascular Research), Partner Site Munich Heart Alliance, University Hospital, LMU, Munich, Germany; ^3^Department of Cardiac Surgery, University Hospital, LMU, Munich, Germany

## Abstract

**Background:**

The impact of devices for vessel closure on the safety and efficacy of cannula removal in VA-ECMO patients is unknown.

**Methods:**

We retrospectively analyzed 180 consecutive patients weaned from VA-ECMO after cardiac arrest or cardiogenic shock from January 2012 to June 2020. In the first period (historical technique group), from January 2012 to December 2018, primary decannulation strategy was manual compression. In the second period (current technique group), from January 2019 to June 2020, decannulation was performed either by a conventional approach with manual compression or by a suture-mediated closure device technique.

**Results:**

A femoral compression system was necessary in 71% of patients in the historical group compared to 39% in the current technique group (*p* < 0.01). Vascular surgery was performed in 12% in the historical cohort and 2% in the current technique cohort, which indicated a clear trend, albeit it did not reach significance (*p* = 0.07).

**Conclusion:**

We illustrated that a suture-mediated closure device technique for VA-ECMO decannulation was feasible, safe, and may have reduced the need of surgical interventions compared to manual compression alone.

## 1. Introduction

Venoarterial extracorporeal membrane oxygenation (ECMO) is a treatment option for patients with ongoing cardiac arrest or severe cardiogenic shock. Nevertheless, mortality and complication rates in these patients remain high and are partially linked to events during the decannulation process. Previous meta-analyses reported major vascular complications including severe bleedings, limb ischemia, compartment syndrome, and extremity amputation in almost 20% of all cases [1]. Another retrospective study could show a lower rate of complications for percutaneous techniques compared to surgical approaches [2]. A first randomized study of Danial et al. found fewer local infections, similar rates of ischemia and sensory motor complication, as well as improved 30-day survival if decannulation was performed percutaneous with manual compression instead of a surgical approach [3]. Dedicated devices for vessel closure including Perclose ProGlide (Abbott Vascular, USA) [4, 5] were developed for improving the process of hemostasis and for further reducing complications. However, the impact of these devices on the safety and efficacy of cannula removal compared to manual compression in ECMO patients is unknown.

## 2. Methods

### 2.1. Patients

We retrospectively analyzed 180 consecutive patients weaned from VA-ECMO after cardiac arrest or cardiogenic shock from January 2012 to June 2020. In the first period (historical technique group), from January 2012 to December 2018, primary decannulation strategy was manual compression. In the second period (current technique group), from January 2019 to June 2020, decannulation was performed at the discretion of the attending physician, either by a conventional approach with manual compression or by a suture-mediated closure device technique (Perclose ProGlide, Abbott Vascular, USA) as previously reported [4]. In compliance with the Declaration of Helsinki and German data protection laws, all patients in this analysis were treated in cardiac intensive care unit (ICU) of Ludwig Maximilian University Hospital and included in a registry (LMUshock, WHO registry number: DRKS00015860, local IRB number: 18-001).

### 2.2. Study Endpoints

Study endpoints were use of a femoral compression device (FemoStop, St. Jude Medical, USA) and surgery for decannulation.

### 2.3. Procedure

Initial VA-ECMO cannula implantation was guided by fluoroscopy or ultrasound to avoid a puncture distally from femoral artery bifurcation. Heavy calcifications at the puncture site were a contraindication for VA-ECMO cannula implantation per se. Puncture sites for arterial (15-19 Fr) and venous cannula (21-24 Fr) were proximal femoral artery and vein. Antegrade perfusion sheath (8 Fr) was inserted into the superficial femoral artery.

Decannulation was performed at bedside in our intensive care unit to avoid transportation to the catheterization laboratory. In case of focal calcification, which prevented the first attempt of closure device ProGlide application, another attempt was undertaken with a different angle of insertion. Absence or unavailability of the two suture device trained physicians was the primary reason for abstaining from the suture-mediated closure device. Anticoagulation was stopped 4 hours before planned decannulation. In the conventional approach, VA-ECMO was stopped and both cannulas were removed. We applied manual compression on cannulation sites for 30–45 minutes until hemostasis. In absence of any bleeding, only a standard pressure bandage was applied. In case of persistent bleeding, a femoral compression device (FemoStop, St. Jude Medical, USA) was used to sustain hemostasis. Compression pressure was gradually reduced from 20 mmHg above systolic blood pressure by 20 mmHg every 15 minutes. By reaching a pressure below 30 mmHg, compression bandage was applied for further 12 hours. Alternatively, we used a suture-mediated closure device technique according to our standardized protocol as published previously [4]. In brief, after clamping and cutting the arterial cannula, a hemostasis valve Y connector (Merit Angioplasty Pack™) was inserted into the proximal arterial cannula. A standard 0.035 inch guidewire was then advanced into the hemostasis valve. After removal of the arterial cannula, two suture-mediated closure devices were applied to achieve vessel closure. After final removal of the venous and antegrade perfusion sheath, manual compression was continued for at least 5 minutes and puncture site covered by pressure bandage for 12 hours.

### 2.4. Data Collection

Demographic, procedural, and outcome data were obtained from review of our LMUshock registry (WHO registry number: DRKS00015860, local IRB number: 18-001).

### 2.5. Statistical Analysis

Statistical analysis was performed using R (version 4.0.1, the R foundation, Vienna, Austria). Normally distributed continuous variables were reported as mean with standard deviation and nonnormally distributed continuous variables as median with interquartile ranges (25^th^ and 75^th^ percentile). The *t*-test for normally distributed continuous variables and Mann–Whitney *U* test for nonnormally distributed continuous variables were used to compare groups. Categorical variables were reported as absolute numbers and percentages, and the chi-square test or Fisher's exact test was utilized for comparison. All tests were 2-tailed, and *p* values <0.05 were considered as significant.

## 3. Results

### 3.1. Baseline Characteristics

**Figure 1:**
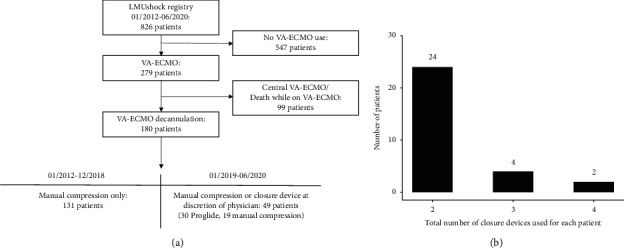
(a) Graphical overview of patient selection. (b) Total number of closure devices used for each patient in the suture-mediated closure device technique group.

In this retrospective analysis, we investigated VA-ECMO decannulation either by manual compression or use of a suture-mediated closure device in 180 patients. Our patient cohort was divided into two groups: a historical group with manual compression from January 2012 to December 2018 (*n* = 131) and a current technique group from January 2019 to June 2020 (*n* = 49). In this latter group, decannulation was performed at the discretion of the attending physician, either by manual compression (*n* = 19) or with a suture-mediated closure device technique (*n* = 30) (Figure 1(a)) [4]. The median number of closure devices used for each patient was 2 in the suture-mediated closure device technique group (Figure 1(b)). No additional suture device type despite Perclose ProGlide was used. All baseline characteristics are given in Table 1.

### 3.2. ICU Parameters

Mean survival after venoarterial ECMO (SAVE) score was about −9 (historical: −8.3; current: −9.0), and mechanical ventilation was applied in almost all patients with VA-ECMO therapy (historical: 93%; current: 98%). Median time of ECMO therapy was 4.2 days in the historical cohort and 3.9 days in our current technique cohort. Almost all patients received therapeutic anticoagulation treatment with unfractionated heparin (historical: 97%; current: 98%). Platelet count was 62 × 10^9^/L in both groups. In half of all cases, dual antiplatelet therapy was applied, predominantly with potent platelet inhibitors (Table 1, all ICU characteristics).

### 3.3. Decannulation Success

**Figure 2:**
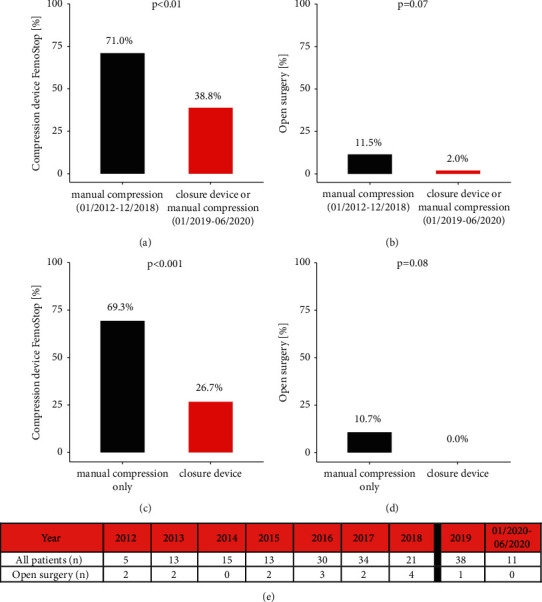
(a) Use of a femoral compression device and (b) switch to open surgery in our historical cohort (manual compression) compared to our current technique (ProGlide or manual compression). (c) Use of a femoral compression device and (d) switch to open surgery in patients with manual compression compared to patients undergoing suture device mediated decannulation (time period-independent). (e) Rates of open surgery throughout the whole study period.

A femoral compression system was necessary in 71% of patients in the historical group compared to 39% in the current technique group (*p* < 0.01) (Table 1, Figure 2(a)). In patients who were decannulated using a suture-mediated closure device, a femoral compression system was only necessary in 8 patients (27%). Vascular surgery was performed in 12% in the historical cohort and 2% in the current technique cohort, which indicates a clear trend, albeit it did not reach significance (*p* = 0.07) (Table 1; Figures 2(b) and 2(e)).

Focusing solely on the type of the decannulation technique, the femoral compression system was applied to 69% of patients without usage of the suture device compared to 27% of patients with suture device decannulation (*p* < 0.001, Figure 2(c)). Furthermore, surgical intervention was required in 11% of patients without usage of suture device. No patient with suture device decannulation required open surgery (*p* = 0.08, Figure 2(d)).

Concerning potential loss of hemoglobin due to bleedings in the process of decannulation, we could not observe a difference in hemoglobin gain after decannulation (historical: 0.40 ± 1.09 g/dL; current: 0.41 ± 1.28 g/dL, *p* = 0.96) and red blood cell transfusions (historical: 1.73 ± 2.50; current: 1.55 ± 2.30, *p* = 0.67). Furthermore, no amputation was necessary due to decannulation. Decannulation with concomitant implanted Impella device, which could in turn increase bleeding risk, was performed in four patients, which all belonged to the historical group. Two of those patients required surgical intervention. No IABP device was used in our cohort.

## 4. Discussion

A first pioneering study has indicated that removal of VA-ECMO cannulas with manual compression might be superior to surgical removal with respect to local infections, ischemia, and even survival [3]. Several successive case series have demonstrated that decannulation could even be improved if closure devices like Perclose ProGlide (Abbott Vascular, USA) or MANTA (Teleflex, USA) were applied [4, 6, 7].

In this retrospective study, we compared a historical cohort of manual compression strategy with an updated current technique of decannulation which included the use of a suture-mediated closure device technique at the discretion of the attending physician. We showed a clear trend towards a reduced need for surgical intervention in the current technique group. Beyond pure procedural success, the suture-mediated closure device technique in our current approach provided significantly higher rates of initial successful hemostasis and therefore circumvented the additional use of a femoral compression system (FemoStop, St. Jude Medical, USA).

This trial on percutaneous VA-ECMO decannulation was limited by its small patient cohort, its retrospective design, and the lack of randomization. Furthermore, the unequal size of both groups could lead to unequal variances, thereby affecting the assumption of equal variances for the *t*-test but not for the chi-square test. The comparison between a historical technique and our updated current approach allowed no direct comparison between manual compression and the suture-mediated closure device technique but reflected a real-life clinical scenario.

## 5. Conclusion

In this study, we illustrated that a suture-mediated closure device technique for VA-ECMO decannulation was feasible, safe, and may have reduced the need of surgical interventions compared to manual compression alone. The implantation of suture-mediated closure devices was not associated with any negative side effects. Larger randomized trials are needed to confirm our findings and generate a new standard for VA-ECMO decannulation.

## Figures and Tables

**Table 1:** Characteristics and endpoints for the historical vs. current technique group.

Variables	Manual compression (*n* = 131)	Closure device or manual compression (*n* = 49)	*P* value	Statistical test
Age, years (SD)	55.4 (12.6)	58.8 (10.9)	0.10	*t*-test
Male gender, *n* (%)	106 (80.9)	37 (75.5)	0.55	Chi-square test
Previous PCI, *n* (%)	39 (29.8)	13 (26.5)	0.81	Chi-square test
Previous CABG, *n* (%)	6 (4.6)	4 (8.2)	0.57	Chi-square test
Atrial fibrillation, *n* (%)	30 (22.9)	7 (14.3)	0.29	Chi-square test
Previous stroke, *n* (%)	13 (9.9)	6 (12.2)	0.86	Chi-square test
Peripheral artery disease, *n* (%)	3 (2.3)	2 (4.1)	0.89	Chi-square test
Smoker, *n* (%)			0.47	Chi-square test
Active smoker	43 (32.8)	19 (38.8)		
Former smoker	19 (14.5)	4 (8.2)		
Never smoked	69 (52.7)	26 (53.1)		
Hypertension, *n* (%)	66 (50.4)	35 (71.4)	0.02	Chi-square test
High cholesterol, *n* (%)	45 (34.4)	20 (40.8)	0.53	Chi-square test
Diabetes, *n* (%)	23 (17.6)	17 (34.7)	0.02	Chi-square test
Cardiac arrest, *n* (%)	86 (65.6)	36 (73.5)	0.41	Chi-square test
Out of hospital cardiac arrest, *n* (%)	45 (34.4)	13 (26.5)	0.41	Chi-square test
Cause of cardiogenic shock or cardiac arrest, *n* (%)			0.24	Chi-square test
Arrhythmia	7 (5.3)	1 (2.0)		
Cardiomyopathy	23 (17.6)	5 (10.2)		
Intoxication	0 (0.0)	1 (2.0)		
Pulmonary embolism	3 (2.3)	0 (0.0)		
Myocarditis	8 (6.1)	4 (8.2)		
NSTEMI	24 (18.3)	14 (28.6)		
Others	2 (1.5)	1 (2.0)		
Septic shock	1 (0.8)	2 (4.1)		
STEMI	60 (45.8)	21 (42.9)		
Valvular	3 (2.3)	0 (0.0)		
First lactate on ICU, median (IQR)	7.9 (3.6, 9.6)	7.3 (4.0, 9.6)	0.86	Mann–Whitney *U* test
First GFR on ICU, median (IQR)	45.0 (33.0, 56.0)	42.0 (31.0, 56.0)	0.67	Mann–Whitney *U* test
ASA, *n* (%)	88 (67.2)	38 (77.6)	0.24	Chi-square test
Clopidogrel, *n* (%)	4 (3.1)	2 (4.1)	1.00	Chi-square test
Prasugrel, *n* (%)	57 (43.5)	23 (46.9)	0.81	Chi-square test
Ticagrelor, *n* (%)	16 (12.2)	2 (4.1)	0.18	Chi-square test
UFH, *n* (%)	127 (96.9)	48 (98.0)	1.00	Chi-square test
SAVE score, mean (SD)	−8.3 (5.5)	-9.0 (4.9)	0.49	*t*-test
Duration ECMO therapy in days, median (IQR)	4.2 (3.0, 6.0)	3.9 (2.7, 7.0)	0.87	Mann–Whitney *U* test
Mean arterial sheath size, Fr (SD)	16.73 (1.37)	16.45 (1.00)	0.17	*t*-test
Mean venous sheath size, Fr (SD)	23.00 (1.45)	22.70 (0.93)	0.20	*t*-test
Platelet count at decannulation per 10^9^/L, median (IQR)	71.0 (52.0, 105.5)	72.5 (45.8, 101.2)	0.88	Mann–Whitney *U* test
INR at decannulation, median (IQR)	1.2 (1.1, 1.4)	1.1 (1.0, 1.2)	0.06	Mann–Whitney *U* test
PTT at decannulation in sec, median (IQR)	37.0 (30.0, 47.8)	37.0 (28.0, 51.2)	0.74	Mann–Whitney *U* test
Use of ProGlide decannulation system, *n* (%)	0 (0.0)	30 (61.2)	<0.01	Chi-square test
Use of femoral compression system FemoStop for decannulation, *n* (%)	93 (71.0)	19 (38.8)	<0.01	Chi-square test
Open surgery on decannulation, *n* (%)	15 (11.5)	1 (2.0)	0.07	Fisher's exact test

All values are presented as mean and standard deviation (SD), median and interquartile range (IQR), or percent of total, respectively. PCI, percutaneous coronary intervention; CABG, coronary artery bypass graft; ICU, intensive care unit; GFR, glomerular filtration rate; ASA, acetylsalicylic acid; UFH, unfractionated heparin; SAVE score, survival after venoarterial ECMO score; PTT, partial thromboplastin time; INR, international normalized ratio.

## Data Availability

The data used to support the findings of this study are available from the corresponding author upon request.

## Abbreviations

VA-ECMO: Venoarterial extracorporeal membrane oxygenation
ICU: Intensive care unit
PCI: Percutaneous coronary intervention.
